# Exploring the Role of Virtual Reality in Labor Induction: A Narrative Review of Benefits, Challenges, and Future Directions

**DOI:** 10.1155/jp/6419145

**Published:** 2026-06-17

**Authors:** Eliannore Boutros, Mohamad Abdelkhalik, Layal Alboutary, Maria Ibrahim, Jennifer Khoury

**Affiliations:** ^1^ Gilbert and Rose-Marie Chagoury School of Medicine, Lebanese American University, Byblos, Lebanon, lau.edu.lb

**Keywords:** anxiety, induction of labor, pain management, virtual reality

## Abstract

**Background:**

Induction of labor (IOL) is a frequent strategy to improve maternal and fetal outcomes, but both pharmacological and mechanical approaches have hazards, including uterine hyperstimulation and fetal discomfort.

**Objective:**

This research investigates the potential use of virtual reality (VR) for labor induction, focusing on pain, anxiety, maternal satisfaction, labor progression, and newborn outcomes.

**Methods:**

A review of research, randomized trials, and meta‐analyses was conducted to evaluate VR’s role in labor, considering maternal experiences, physiological responses, and clinical outcomes.

**Results:**

Evidence shows that VR decreases pain and anxiety during IOL while increasing maternal satisfaction; its effect on labor length is inconsistent. No significant influence on newborn outcomes, such as Apgar scores, was seen. Pain severity, duration of usage, past VR exposure, and individual preferences all have an impact on VR adoption.

**Conclusion:**

VR shows potential as an auxiliary to obstetric treatment, improving maternal comfort and emotional well‐being. However, further large‐scale trials are required to validate its therapeutic advantages and safety, particularly in newborn outcomes.

## 1. Introduction

During the last decade, the term *virtual reality* (VR) has gained widespread recognition, evolving from early concepts in the mid‐1980s into a technology increasingly applied across multiple domains. VR is an immersive, interactive, multisensory, viewer‐centered simulation environment created through computer‐generated imagery, allowing users to engage with a three‐dimensional, virtual world in a highly realistic manner [1, 2]. In medicine, VR has been applied for patient rehabilitation and as a key component in the training of medical students [3]. The core components of VR technology include a headset, which displays the virtual environment and tracks head movements, sensors, and cameras that monitor user actions and spatial orientation, and input devices such as controllers or gloves to interact with the virtual space [4]. Audio components further enhance the virtual space [4]. Together, these components enable users to explore virtual environments in a way that closely mimics real‐world interactions.

Induction of labor (IOL) is one of the most common procedures performed in obstetrics and one of the fastest‐growing medical procedures [5]. It involves initiating uterine contractions to deliver the fetus before the natural onset of labor. According to the American College of Obstetrics and Gynecology (ACOG) Practice Bulletin No 107, IOL is indicated when the benefits of delivery outweigh the risks of continuing the pregnancy, such as in post‐term pregnancy, suspected intrauterine growth restriction, pre‐labor rupture of membranes, hypertensive disorders of pregnancy, and maternal diabetes complicating pregnancy [6]. In other cases, it may be performed electively. Multiple methods exist for IOL, including pharmacologic approaches and mechanical interventions, often requiring cervical ripening to soften and dilate an unfavorable cervix, reduce time to delivery, and lower the risk of failed induction [7].

While traditional IOL methods are effective, they carry inherent risks, including uterine hyperstimulation, fetal distress, and increased likelihood of operative delivery [8, 9]. Given these physiological and psychological challenges, non‐pharmacologic adjuncts such as VR may improve maternal experience without altering uterine contractility. By targeting maternal perception of pain and anxiety, VR provides distraction, relaxation, and a sense of control during labor, rather than directly modifying uterine physiology. This approach complements standard induction techniques, enhancing maternal satisfaction and overall childbirth experience.

VR has shown promising applications in diverse medical contexts, including pain management, procedural distraction, neurorehabilitation, and medical education [10, 11]. In obstetrics, VR has been explored as a supportive tool to optimize the labor experience by reducing perceived pain and anxiety, enhancing maternal engagement, and improving maternal satisfaction with the childbirth process. This paper aims to synthesize the existing literature on VR during labor induction, highlighting its potential role as a non‐pharmacologic adjunct to support maternal well‐being during IOL.

## 2. Methods

### 2.1. Study Design

This is a narrative review with systematic search elements, designed to provide a comprehensive overview of VR use in labor while maintaining transparency and reproducibility in the literature search process.

### 2.2. Search Strategy

The literature search was initially conducted in January 2025 and updated twice in October 2025 and March 2026 to include newly published studies. The following electronic databases were searched: PubMed, SciELO, CINAHL, Web of Science, and ScienceDirect.

The search strategy used Boolean operators and included the following terms: (“virtual reality” or “VR”) AND (“labor” OR “childbirth” OR “obstetrics” OR “labor pain” OR “induction of labor”).

Search terms were adapted as needed for each database.

### 2.3. Inclusion Criteria

Studies were included if they met the following criteria:

1. Published in English.
2. Published between January 2000 and March 2026 (data last updated in March 2026).
3. Addressed the use of VR in labor, childbirth, or obstetric care.
4. Included study designs such as randomized controlled trials (RCTs), cohort studies, case–control studies, cross‐sectional studies, and relevant review articles.

Studies were excluded if they were not relevant to the topic, not available in English, or published prior to 2000 unless considered of significant historical relevance.

### 2.4. Study selection

Two independent reviewers conducted the screening process. All retrieved records were imported in Zotero, where duplicates were identified and removed.

The remaining studies were screened using Rayyan. Titles and abstracts were independently reviewed based on eligibility criteria. Full‐text articles were then assessed for final inclusion.

Discrepancies between reviewers were resolved through discussion and consensus.

**Figure 1:**
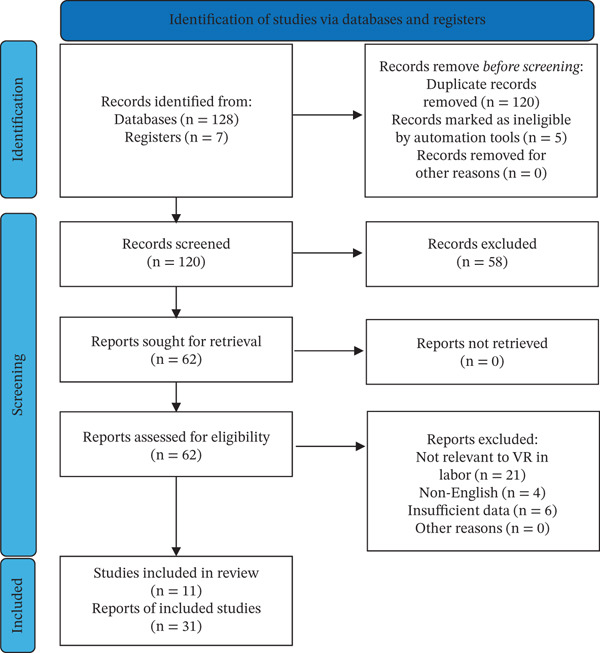
PRISMA Flow Diagram of Study Selection for Virtual Reality Use in Labor. Total included studies (n = 11) represent the final sample for qualitative synthesis.

A PRISMA‐style flow diagram was used to illustrate the study selection process and summarized in Figure 1.

### 2.5. Data Extraction

Data extraction was performed independently by two reviewers using a standardized approach. Extracted variables included:

▪ Study design
▪ Sample size
▪ Type of VR intervention
▪ Clinical application
▪ Outcomes (e.g., pain scores, anxiety levels, maternal satisfaction)
▪ Key findings and limitations

### 2.6. Data Synthesis

Because of heterogeneity in study designs, interventions, and outcome measures, a quantitative meta‐analysis was not performed. Studies were synthesized narratively, focusing on trends in clinical effectiveness, patient experience, and implementation considerations.

## 3. Results and Discussion

### 3.1. Traditional Methods of Labor Induction

IOL can be achieved through pharmacological or mechanical methods. Pharmacologic approaches, including prostaglandins (misoprostol, dinoprostone) and oxytocin, are commonly used to ripen the cervix and stimulate uterine contractions [10, 12]. Mechanical interventions, such as membrane sweeping, amniotomy, and balloon catheters, promote cervical dilatation, and labor onset [11, 13]. Both approaches are effective but carry risks, including uterine hyperstimulation, tachysystole, fetal distress, and rarely, uterine rupture [14]. Given these potential complications and the psychological burden of labor, non‐pharmacologic adjuncts such as VR may improve maternal experience without altering uterine contractility.

### 3.2. Introduction to VR in Healthcare

VR has evolved from early specialized applications to an accessible technology with potential benefits in healthcare. In clinical settings, VR is primarily used to reduce pain, anxiety, and stress by immersing patients in computer‐generated, multisensory environments [15, 16]. VR has been shown to decrease acute procedural pain in pediatric populations and during painful interventions such as bone marrow biopsy or lumbar puncture [17, 18]. Mechanistically, VR works through attentional distraction, modulation of autonomic responses (e.g., heart rate and blood pressure), and interruption of the stress–pain feedback loop, which are particularly relevant for laboring women experiencing nociceptive and psychological stress [18, 19]. By engaging patients in immersive or interactive experiences, VR can serve as a non‐pharmacologic adjunct to support maternal comfort and well‐being during labor, without directly altering uterine contractility.

### 3.3. Types and Mechanisms of VR Interventions in Labor

VR should not be considered a uniform intervention, as its clinical effects depend on both the level of immersion and the nature of the content delivered. VR interventions in labor can be classified based on degree of immersion, type of content, and intended therapeutic effect.

In terms of immersion, VR may be immersive, involving head‐mounted displays that fully engage the user in a three‐dimensional environment, or non‐immersive, where interaction occurs through standard screens with limited sensory engagement.

Immersive VR is associated with greater sensory isolation and stronger distraction effects, as reported in studies assessing pain and anxiety during labor [20–23].

Content‐wise, VR applications can be categorized into the following: (1) Passive relaxation experiences, such as nature scenes or guided meditation aimed at reducing stress and anxiety [24, 25]. (2) Active distraction‐based environments, including interactive games that divert attention away from nociceptive stimuli [24]. (3) Emotionally engaging content, such as fetal ultrasound visualization, which may enhance maternal‐fetal bonding and positively influence perception of labor (Atkin et al., 2021) [22].

The therapeutic effects of VR are thought to arise through several mechanisms. First, attentional distraction reduces the cognitive processing of pain signals [20, 21, 26]. Second, through gate control mechanisms, VR may modulate pain perception, whereby competing sensory input inhibits nociceptive transmission [27, 28]. Third, VR has been associated with autonomic modulation, including reductions in heart rate and blood pressure, reflecting decreased sympathetic activation transmission [21, 27]. Finally, by reducing anxiety, VR may interrupt the stress–pain feedback loop responsible for amplifying pain perception during labor [23, 26].

This framework provides a basis for interpreting the variability in clinical outcomes reported across studies, as differences in VR type, content, and timing of use may significantly reduce effectiveness [20, 28, 29].

### 3.4. Empirical Evidence on the Use of VR in Labor Induction

Several studies have investigated the effectiveness of VR in labor induction, particularly regarding pain reduction, anxiety alleviation, and maternal satisfaction, with some studies also assessing labor progression and neonatal outcomes.

#### 3.4.1. Pain Reduction

VR interventions have consistently demonstrated benefits in reducing labor pain. Twelve studies within the cited meta‐analysis including 1095 participants reported that VR decreased the duration of the active phase by approximately 25.6 min and reduced pain scores by 1.81 points on a standard 10‐point Visual Analog Scale (VAS) [20].

Multiple RCTs corroborated these findings. An RCT with 40 women showed a significant reduction in pain (–0.52 on VAS) and lower maternal heart rate in the VR group compared with an increase in pain (+0.58) in the control group [21]. Another trial demonstrated lower heart rates (79.86 vs. 85.57 beats per minute) and mean arterial pressures (88.78 vs. 92.61 mmHg), along with lower VAS pain scores (6.14 vs. 7.61) in women using VR [27].

Specific VR content may influence pain outcomes. For example, Akin et al. used fetal ultrasound visualization through VR glasses, resulting in significantly lower VAS pain scores and reduced anxiety [22]. Small‐sample studies (*n* = 8) also reported decreased pain and lower epidural use with VR compared with national averages [23]. Pain reduction was observed across cervical dilation stages, with meta‐analytic effect sizes ranging from mean difference (MD) −0.43 (≤ 4 cm) to standardized mean difference (SMD) −1.91 (≥ 9 cm) [28].

#### 3.4.2. Anxiety Reduction

VR also reduces labor‐related anxiety. Meta‐analyses reported significant reductions in anxiety scores during labor for VR users (SMD of −1.15 and −1.08) [26, 28]. The Perinatal Anxiety Screening Scale (PASS) confirmed lower anxiety levels 2 h post‐labor in women who used VR, particularly at 9 cm cervical dilation [22]. Quantitative studies further support this effect, showing that VR interventions helped women relax and manage anxiety during labor [23, 25].

#### 3.4.3. Physiological Response

VR may modulate physiological stress responses during labor. Lower maternal heart rate and mean arterial pressure were documented in multiple studies, reflecting reduced sympathetic activation and supporting a mechanism of autonomic modulation [21, 26, 27].

#### 3.4.4. Labor Duration

Evidence on labor progression is mixed. Some meta‐analyses reported no significant impact of VR on the first and second stages of labor [20, 27]. In contrast, Özer et al. found significant reductions in the first (SMD −0.53) and second (MD −0.39) stages among VR users [28]. Differences may reflect VR type, exposure duration, labor stage, or sample size.

#### 3.4.5. Maternal Satisfaction

Maternal satisfaction with VR is consistently high. A meta‐analysis reported a 32% increase in satisfaction (relative risk [RR] = 1.32) [20]. Individual studies confirmed these findings, including Baradwan et al. (MD 15.58) and Özer et al. (mean increase 11.24 points) [26, 28]. Carus et al. found a mean satisfaction score of 87.7 ± 12.9 out of 100 [30].

Qualitative research highlights user preferences: women favored guided meditation VR over interactive games but appreciated both, and emphasized VR as a complementary method, not a substitute for standard analgesia [25]. VR comfort (controllers, goggles, seating) and prior exposure also influenced acceptability [25, 31].

#### 3.4.6. Neonatal Outcomes

Current evidence on neonatal outcomes is limited. A small RCT showed no significant differences in Apgar scores or birth weights using VR and control groups, suggesting VR interventions during labor do not adversely affect neonates [29].

The key characteristics and outcomes of the included studies are summarized in Table 1, providing a detailed overview of VR interventions, study design, sample size, and measured maternal and neonatal outcomes.

**Table 1:** Summarizing findings of the studies.

Outcome domain	Author (year)	Study design	Sample size	VR type (immersion/content)	Outcome domain	Main findings
Pain and anxiety outcomes	Xu et al. (2022) [20]	Systematic review and meta‐analysis	1095	Immersive (mixed content)	Pain, anxiety	Reduced pain and anxiety; improved maternal satisfaction
	Baradwan et al. (2022) [26]	Meta‐analysis	466	Mixed	Pain, anxiety	Significant reduction in pain and anxiety
	Akin et al. (2021) [22]	Randomized controlled trial	100	Immersive (fetal VR)	Pain, anxiety	Reduced pain and anxiety; improved childbirth perception
	Cole (2023) [23]	Quality improvement study	8	Immersive	Pain	Reduced pain and epidural use
	Özer et al. (2024) [28]	Systematic review and meta‐analysis	756	Mixed	Pain, anxiety	Significant reductions in pain and anxiety
	Musters et al. (2023) [24]	Qualitative study	24	Passive vs. active VR	Pain	Pain reduction; preference for guided relaxation
	Massov et al. (2023) [27]	Qualitative study	14	Immersive	Pain, anxiety	Reduced pain and anxiety; improved relaxation

Physiological outcomes	Massov et al. (2023) [27]	Intervention study	14	Immersive	Physiologic	Reduced heart rate and mean arterial pressure

Labor progression	Xu et al. (2022) [20]	Meta‐analysis	1095	Mixed	Labor duration	Shortened active labor; no effect on stages
	Özer et al. (2024) [28]	Meta‐analysis	756	Mixed	Labor duration	Reduced duration of first and second stages

Maternal satisfaction and experience	Carus et al. (2022) [30]	Randomized controlled trial	42	Immersive	Satisfaction	High maternal satisfaction scores
	Musters et al. (2023) [24]	Qualitative study	24	Passive vs. active VR	Satisfaction	Preference for passive VR
	Leugenhaege et al. (2024) [25]	Qualitative study	10	Passive immersive	Experience	Preference for calming VR; some discomfort reported
	Cowles et al. (2019) [31]	Observational study	20	Immersive	Experience	Less preferred at high pain levels and nighttime
	Massov et al. (2023) [27]	Intervention study	14	Immersive	Experience	Improved relaxation and emotional experience

Neonatal outcomes	Xie and Zeng (2023) [29]	Randomized controlled trial	200	Immersive	Neonatal	No significant effect on Apgar scores or birth weight

## 4. Limitations and Future Direction

Despite promising findings, several limitations exist. Many studies have small sample sizes, variable experimental designs, and heterogeneity in VR type, content, and duration. Conflicting evidence regarding labor progression highlights the need for larger, standardized trials. Additionally, neonatal outcomes are underreported. Future research should use validated quantitative and qualitative tools to assess maternal pain, anxiety, and satisfaction, while systematically evaluating neonatal safety outcomes.

To advance the clinical application of VR in labor induction, future research should focus on developing standardized VR protocols, including optimal timing, duration, and content of exposure. Integration of VR into routine induction workflows should be evaluated for feasibility and effectiveness compared to the standard of care approach. RCTs adequately powered to assess neonatal outcomes are needed to establish safety and efficacy for the fetus. Additionally, cost‐effectiveness analysis would support broader adoption, while prenatal VR familiarization programs could improve maternal acceptance and comfort during labor.

## 5. Conclusion

VR has the potential to be a beneficial tool in labor management, particularly during induction, by reducing pain and anxiety and increasing maternal satisfaction. While some studies suggest possible effects on labor progression, the evidence remains inconsistent, highlighting the need for further well‐designed research. VR should be introduced as a supportive tool alongside established pain management strategies, with attention to individual patient preferences and comfort. Although maternal benefits are increasingly recognized, the impact on neonatal outcomes remains insufficient in the studies, and additional research is required to confirm fetal safety.

## Author Contributions

Eliannore Boutros, Mohamad Abdelkhalik, Layal Alboutary, and Maria Ibrahim have contributed to the work equally and should be regarded as co‐first authors.

## Funding

No funding was received for this manuscript.

## Conflicts of Interest

The authors declare no conflicts of interest.

## Data Availability

The data that support the findings of this study are available on request from the corresponding author. The data are not publicly available due to privacy or ethical restrictions.
